# Synthesis, Characterization, and Electrochemical Behavior of Layered Vanadium Nitride MXene

**DOI:** 10.1021/acsnano.5c14516

**Published:** 2025-09-20

**Authors:** Bright Ngozichukwu, Niels Kubitza, Laura Hoagland, Christina S. Birkel, Abdoulaye Djire

**Affiliations:** † Artie McFerrin Department of Chemical Engineering, 14736 Texas A&M University, College Station, Texas 77843, United States; ‡ Department of Chemistry and Biochemistry, 7864 Technische Universitat Darmstadt, Darmstadt 64287, Germany; § School of Molecular Sciences, Arizona State University, Tempe AZ-85282, United States; ∥ Department of Materials Science & Engineering, Texas A&M University, College Station, Texas 77843, United States

**Keywords:** vanadium nitride, MNene, MXene, molten-salt, energy storage, supercapacitor

## Abstract

MXenes have been extensively studied for over a decade, with numerous compositions successfully synthesized. However, the top-down synthesis of vanadium nitride (V_2_NT_x_) has remained elusive despite its predicted superior electrochemical performance and stability in aqueous environments. In this work, we demonstrate the synthesis of V_2_NT_x_ MNene by utilizing our oxygen-assisted molten salt etching (O_2_-MSE) method. We additionally demonstrate the versatility of our synthesis approach on the corresponding carbide phase (V_2_CT_x_), confirming its applicability across MAX phases regardless of the X element. Comprehensive structural, physical, chemical, and electrochemical characterizations confirm the MNene’s crystallinity, high surface area, tunable surface chemistry, layered morphology, and superior electrochemical performance compared to its carbide counterpart. As a proof of concept, synthesized V_2_NT_x_ MNene and V_2_CT_x_ MXene were tested as electrodes in an electrochemical device using aqueous electrolytes. The results reveal that the MNene outperforms the carbide in terms of higher capacity, enhanced cycling stability, and better overall performance compared to the carbide phase. For example, in an acidic electrolyte (1 M H_2_SO_4_), V_2_NT_x_ achieved a specific capacity of 123 mAh g^–1^, surpassing the 93 mAh g^–1^ of V_2_CT_x_. Further analysis reveals the enhanced electrochemical performance of V_2_NT_x_ MNene is attributed to the −O and/or −OH surface groups, which undergo more reversible redox reactions in acidic environments compared to alkaline media, which is in contrast to conventional bulk VN (nonlayered) material. In summary, we report the synthesis of layered vanadium MNene via the O_2_-MSE method, demonstrating its stability, electrochemical activity, and surface chemistry that enhances energy storage and conversion.

## Graphical abstract

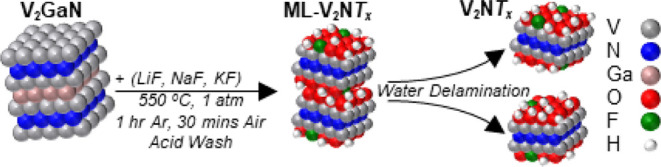

## Introduction

The growing demand for energy storage continues to drive the development of innovative materials capable of storing large amounts of energy within a compact volume while delivering a high power density. One family of materials that shows promise is called MXenes, which are derived from ternary carbides and nitride MAX phases.^1^ M represents a d-block transition metal, A is a main group metal, and X is carbon and/or nitrogen. Three-dimensional MAX phases are typically etched to remove the *A* layer, forming the corresponding two-dimensional MXene.^2,3^ The formula for MXenes is M_n+1_X_n_T_x_, where T_*x*_ indicates surface termination groups (typically O, OH, and F), and *n* is an integer (1–4) that indicates the number of rows for that atom within the 2D layer structure. MXenes are then delaminated to form single- and few-layer structures, which are held together with weak stacking forces,^2^ giving rise to a material with a high surface-area-to-volume ratio, promising electronic conductivity, and extreme tunability.^4–8^ These characteristics are why MXenes are desirable for energy storage applications.^9–14^

The most commonly studied MXene is Ti_3_C_2_T_*x*_. This MXene can be produced by removing aluminum from the MAX starting material Ti_3_AlC_2_ with hydrofluoric acid (HF). However, this synthesis method has been shown to be less effective for producing nitride MXenes (hereafter referred to as MNenes), as M–N bonds are typically broken when etching with HF.^1^ The work by Venkateshalu^2^ attempted to synthesize V_2_NT_x_ MNene using lithium fluoride (LiF) and hydrochloric acid (HCl) to remove the *A* layer from the V_2_AlN MAX phase. While this approach has been previously employed to produce carbide MXenes, their results indicate significant limitations in obtaining V_2_NT_x_ MNene. Their X-ray diffraction (XRD) analysis revealed a persistent presence of the MAX phase, suggesting that the etching process was unsuccessful. Furthermore, the diffraction pattern of the claimed V_2_NT_x_ MNene closely resembles that of the starting V_2_AlN MAX phase, with no presence of the (002) characteristic peak typically observed in MXene. This suggests that the LiF-HCl etching method is not the most effective approach for synthesizing high-quality MNenes.

An alternative synthesis method utilizes the oxygen-assisted molten salt etching (O_2_-MSE) approach, where molten Lewis acid salts such as LiF, sodium fluoride (NaF), and potassium fluoride (KF) are used as etchants.^15–20^ This method has been successfully applied for the synthesis of MNenes while preserving the morphological structure and improving the purity of the MNene. For example, the production of Ti_2_NT_x_ and Ti_4_N_3_T_x_ have been demonstrated using the O_2_-MSE method.^13,21^ Thus, this may be the most viable synthesis approach for the production of new MNene phases.

Vanadium nitrides are of interest for energy storage, in part because their bulk counterpart, VN, has been shown to perform well in aqueous capacitors^8,22–24^ and nonaqueous pseudocapacitors.^25^ Bulk VN performs best in alkaline electrolytes due to the redox activity of vanadium oxynitride (VN_x_O_y_) resulting from the insertion of anions.^8,22^ However, MXenes and MNenes usually perform better in acidic solutions because the termination groups, O and OH,^12,26–29^ react readily with intercalated hydronium ions.^5,30^ Vanadium MNenes could potentially exploit both of these chemistries. Initial calculations show that the theoretical energy storage capacity of a lithium-ion monolayer on V_2_NT_x_ exceeds 900 mAh g^–1^.^31^ However, the influence of termination groups on vanadium MNenes, as it pertains to their charge storage mechanism and capacity, has not been explored previously.

In this work, V_2_NT_x_ and V_2_CT_x_-layered materials were synthesized from their respective V_2_GaN and V_2_GaN MAX phase precursors using the O_2_-MSE method. The nitride MAX phase was produced by reacting bulk vanadium nitride with vanadium and gallium powders, while the carbide MAX phase was synthesized by reacting vanadium, gallium, and carbon powders. The O_2_-MSE process was followed by an acid wash and delamination, and the produced few-layer flakes were made into electrodes and tested in an electrochemical Swagelok cell with acidic and alkaline electrolytes. The electrochemical analysis revealed that V_2_NT_x_ MNene exhibited a specific capacity of 123 mAh g^–1^ at 2 mVs^–1^ in 1 M H_2_SO_4_, higher than that of the 93 mAh g^–1^ carbide counterpart, V_2_CT_x_. This performance is attributed to the oxygen and hydroxyl surface terminations that enhance redox activity. These findings show the significance of vanadium nitride MNenes in energy storage and conversion, as well as related applications.

## Results and Discussion

Prior to the transformation of V_2_GaN to its corresponding MNene, the structure of the as-synthesized MAX phases was confirmed through Rietveld refinement (Figure S1), which revealed high phase purity and crystallinity. The refinement profiles showed excellent agreement between the experimental and calculated patterns. The refinement analysis yielded lattice parameters consistent with previously reported values for these MAX phases. Detailed refined lattice parameters, including the unit cell dimensions and phase fractions, are summarized in Table S1. Both V_2_GaN and V_2_GaC phases were indexed to a hexagonal structure belonging to the *P6*_3_ */mmc* space group, in agreement with literature reports.^17^

Having established the phase identity and structural integrity of the parent MAX materials, we will now focus on the transformation of V_2_GaN to V_2_NT_x_ since this MNene has never been synthesized before. The powder X-ray diffraction pattern presented in Figure 1 (see also Figure S2) provides evidence of the structural transformation of the V_2_GaN MAX phase into V_2_NT_x_ MNene using the oxygen-assisted molten salt etching (O_2_-MSE) method. The emergence of distinct diffraction peaks at 2θ = 18.34°, 25.21°, 29.43°, 33.23°, and 46.12°, observed in the molten salt-treated sample (indicated in purple), shows the formation of complex reaction intermediates during the etching process. These peaks are absent in the pristine MAX phase, as observed in Figure 1a.

**Figure 1:**
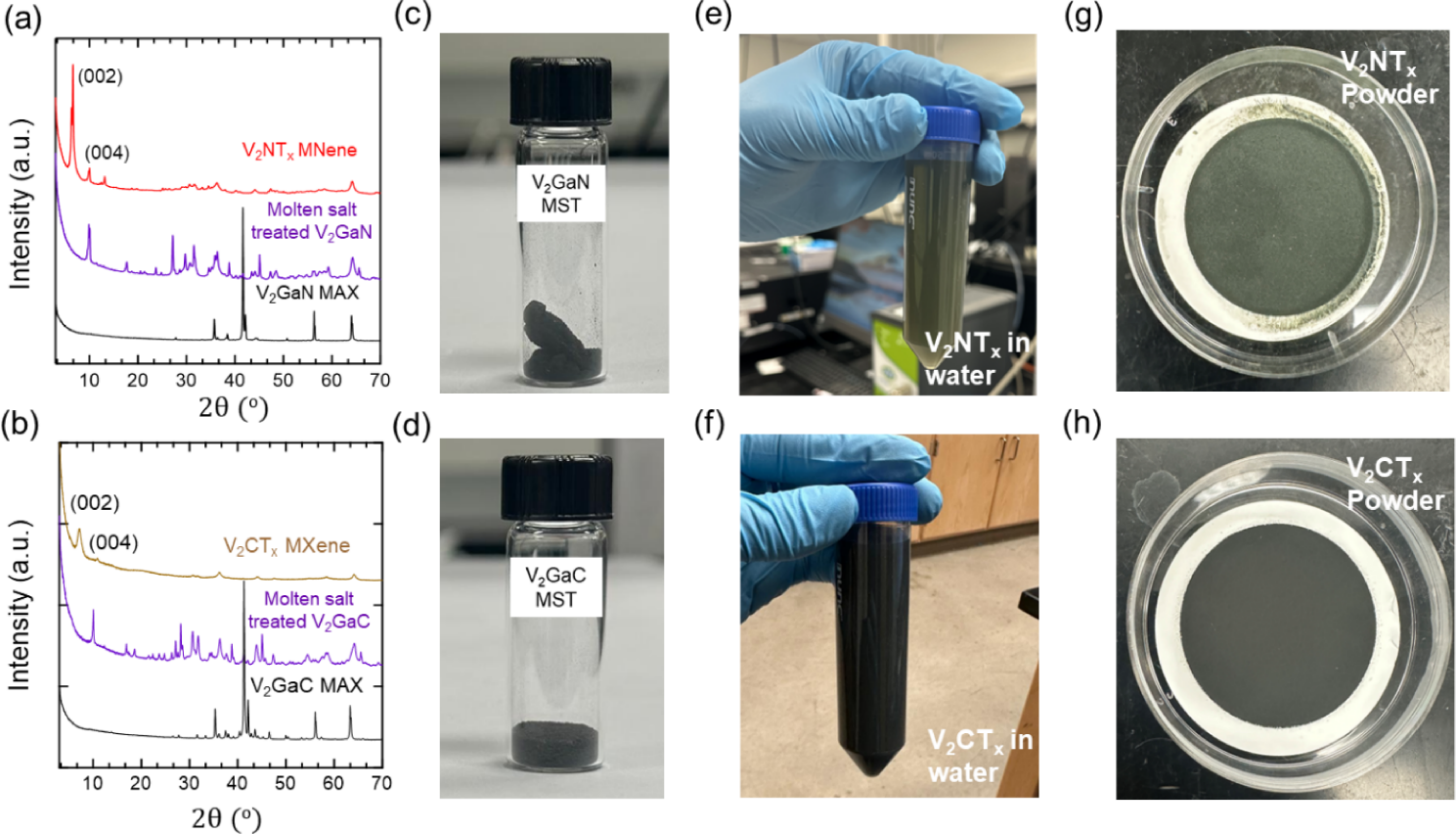
XRD patterns of (a,b) V_2_GaN and V_2_GaC MAX phases (in black), molten salt-treated V_2_GaN and V_2_GaC (in purple), and delaminated V_2_NT_x_ MNene and V_2_CT_x_ MXene (in red and gold, respectively). Photographic images of (c) molten salt-treated V_2_GaN (V_2_GaN-MST) and (d) molten salt-treated V_2_GaC (V_2_GaC-MST) directly from the furnace. Delaminated (e) V_2_NT_x_ MNene and (f) V_2_CT_x_ MXene dispersed in water. Vacuum-filtered (g) V_2_NT_x_ MNene powder and (h) V_2_CT_x_ MXene powder.

Additionally, the disappearance of the characteristic (104) peak of the V_2_GaN MAX phase at 2θ = 42.14° further confirms the transformation of the MAX phase. Notably, we also observe the appearance of an intense peak at 2θ = 9.89°, which is assigned to the (002) basal plane of V_2_NT_x_ MNene (in purple), indicating the etching of gallium and the formation of a multilayered structure after acid washing with formic acid. Further delamination using water reveals a shift of this (002) peak to a lower 2θ = 6.63°, indicating the exfoliation of the multilayer into few-layer or single-layer MNene, as depicted in the red diffraction pattern in Figure 1a. With success in the etching of the V_2_NT_x_ MNene, we turn to the V_2_GaC MAX phase and apply the same O_2_-MSE etching method, as seen in the powder X-ray diffraction pattern in Figure 1b. Attempts have been made to synthesize V_2_CT_x_ using HF solution, but the resulting material was not stable in air or aqueous media.^32^ Applying molten salt to V_2_GaC has never been done before. Similar to the conversion from the V_2_GaN MAX phase to MNene, most of the complex salt peaks in the molten salt-treated stage were removed after the acid wash process. Subsequent delamination results in a diffraction pattern with the (002) peak at 2θ = 5.89° and its reflection at 2θ = 11.29°, confirming the synthesis of V_2_CT_x_ MXene, consistent with previously reported works.^33^ Overall, the O_2_-MSE method is effective for the selective etching of the gallium layer of the MAX phase. The photographic images presented in Figure 1c–h (Figure S1) show the color transformation of the V_2_GaN and V_2_GaC MAX phases to their corresponding MXenes. Both the molten salt-treated V_2_NT_x_ and V_2_CT_x_ show a dark green color, which upon acid washing to remove the complex intermediates and salts and subsequent delamination results in a deep green color for V_2_NT_x_ and a black-green color for V_2_CT_x_, respectively. Previous studies suggest this color shift is indicative of the removal of dissolved vanadium ions originating from intermetallic residual metals, ultimately yielding a stable vanadium-based MXene.^32^

The morphology of the MAX phases and the etched and delaminated V_2_NT_x_ MNene and V_2_CT_x_ MXene were analyzed using scanning electron microscopy (SEM) to evaluate the effectiveness of the O_2_-MSE etching method, as well as to characterize the structural layering of the MNene (Figures 2 and S3). The SEM images of the V_2_GaN and V_2_GaC MAX phase precursors (Figure 2a,e) show a densely packed layered structure, characteristic of the morphology of MAX phases. In contrast, the acid-washed MNene and MXene (Figures 2b,f and S4) exhibit noticeable interlayer spacing and a distinct accordion-like morphology, indicating the etching of the gallium atoms from the corresponding MAX phase structure and the transition to multilayered MNene and MXene. Furthermore, the morphology analysis of the delaminated MNene and MXene shown in Figure 2c,g reveals lateral flake-like few-layer and/or single-layer structures. Elemental distribution mapping, as presented in Figure 2d,h, provides a visualization of the spatial distribution of elements, including V, C/N, and O across the few-layer V_2_NT_x_ MNene and V_2_CT_x_ MXene structures, which confirms the retention of these elements from the parent MAX phases. The presence of oxygen is indicative of surface passivation and functionalization during the etching process.^34^ Figure 2i,j presents the elemental composition analysis of the atomic percentages of V, C, N, Ga, and O. The data show a significant reduction in gallium content by approximately 84% and 80% for the synthesized V_2_NT_x_ MNene and V_2_CT_x_ MXene, respectively, following selective gallium removal from the MAX precursors during etching. Future work will focus on optimizing our etching and washing procedures to achieve a more complete removal of residual A-layer elements, thereby improving material purity. The observed increase in oxygen content is attributed to the etchant and/or etching environment, which introduces O and OH groups, altering the surface chemistry of the MNene and MXene.^21,35,36^ This will be further validated by Fourier-transform infrared (FTIR) spectroscopy and X-ray photoelectron spectroscopy (XPS) analyses.

**Figure 2:**
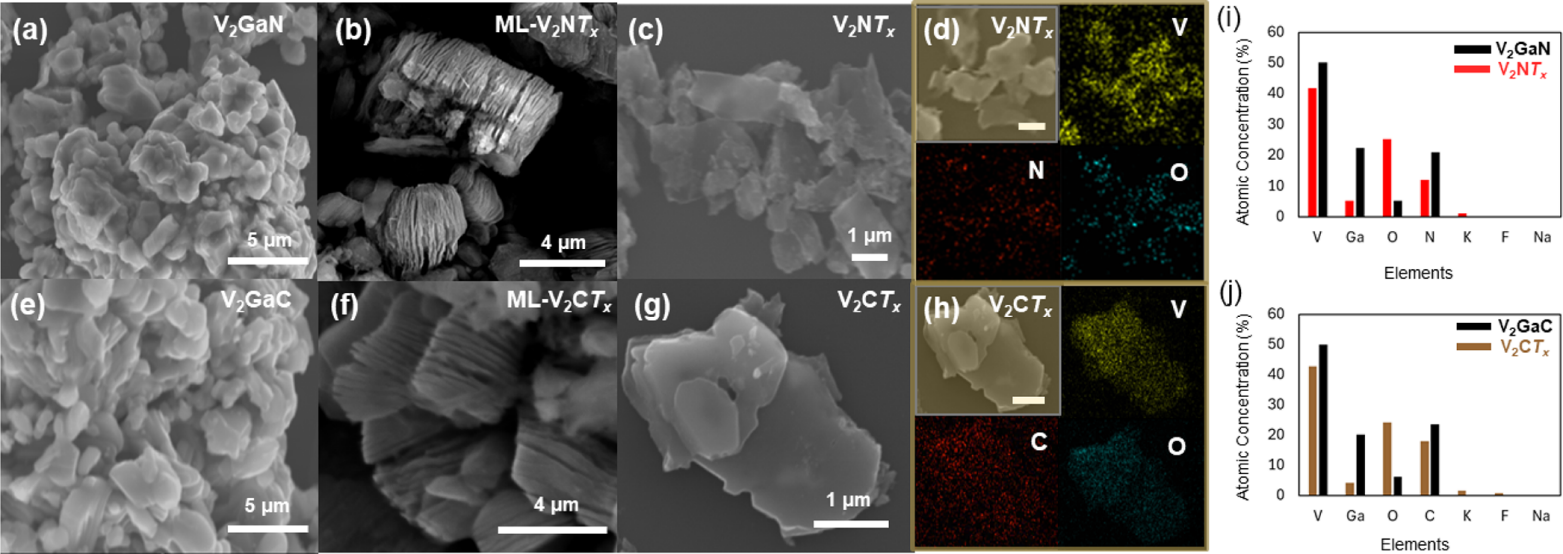
Scanning electron microscopy (SEM) images of (a) V_2_GaN MAX phase and (b) multilayer V_2_NT_x_ MNene after the acid-wash stage. The image reveals a layered morphology with distinct interlayer spacing, evidence of the etching of gallium (A-layer) from the parent MAX phase structure. (c) Delaminated V_2_NT_x_ MNene flakes. (d) EDS elemental mapping for V_2_NT_x_ MNene (scale bar: 1 μm). (e) V_2_GaC MAX phase. (f) Multilayer V_2_CT_x_ MXene. (g) Delaminated V_2_CT_x_ MXene flakes. (h) EDS elemental mapping for V_2_CT_x_ MXene (scale bar: 1 μm). Elemental composition of the (i) V_2_GaN MAX phase and delaminated V_2_NT_x_ MNene phase and (j) V_2_GaC MAX phase and delaminated V_2_CT_x_ MXene phase.

Figure 3 presents low- and high-magnification transmission electron microscopy (TEM) images of the as-synthesized V_2_NT_x_ MNene. The presence of stacked nanosheets, as shown in Figure 3a, confirms the formation of V_2_NT_x_ multilayered MNene, consistent with the SEM results in Figure 2b. The delaminated V_2_NT_x_ reveals a transparent nanosheet-like structure, indicative of a single-layer MNene (Figure 3b). High-resolution TEM (HRTEM), as shown in Figure 3c, provides insight into the atomic arrangement, revealing well-defined features of the V_2_NT_x_ MNene basal plane. Additionally, the selected area electron diffraction (SAED) pattern confirms the presence of hexagonal crystal symmetry (Figure 3c inset), consistent with the previously reported structures of MNenes. Given that TEM analysis of V_2_CT_x_ MXene has been extensively reported,^37–41^ our focus here is primarily on the characterization of V_2_NT_x_ MNene. The surface morphology and thickness of V_2_NT_x_ MNene and V_2_CT_x_ MXene were further examined using atomic force microscopy (AFM), as shown in Figure 3d (see also Figure S5 for the V_2_CT_x_ MXene). The AFM topographic images and corresponding height profiles confirm the exfoliation of these materials into few-to-single-layer flakes. The V_2_NT_x_ MNene flakes exhibit an average thickness of approximately 2.98 nm, indicating a few atomic layers, and possess lateral dimensions on the order of ∼6 μm. Similarly, the V_2_CT_x_ MXene (see Figure S5) flakes show an average thickness of about 1.38 nm, indicating a slightly thinner morphology, with lateral sizes close to 5 μm.

**Figure 3:**
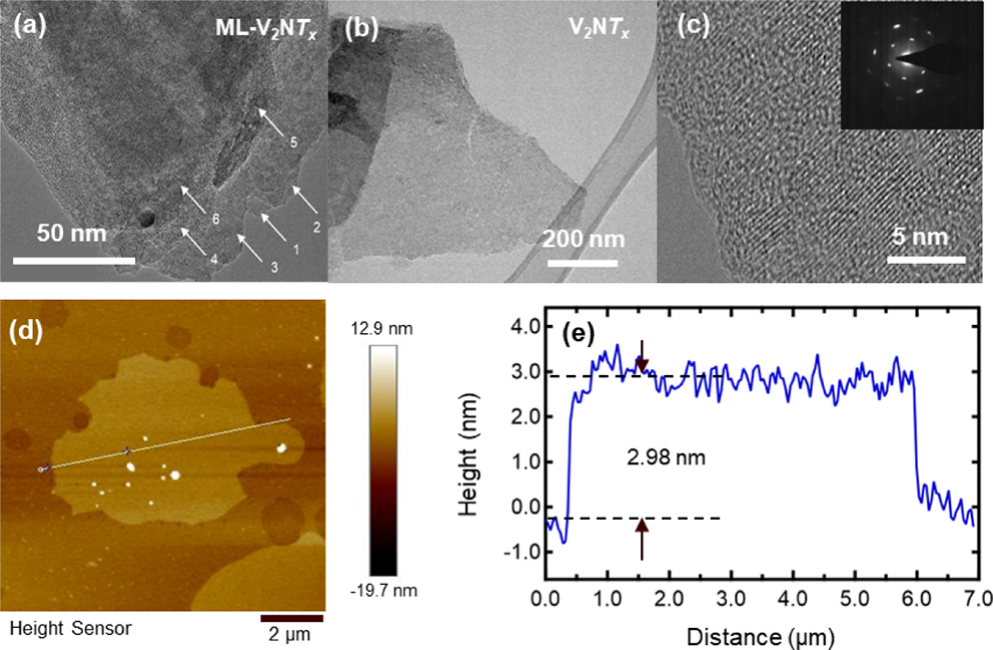
Transmission electron microscopy (TEM) images of (a) nondelaminated (multilayered) V_2_NT_x_ MNene showing the presence of stacked nanoflakes, designated by the numbered arrows. (b) TEM image of delaminated V_2_NT_x_ MNene. (c) High-resolution TEM (HRTEM) image of delaminated V_2_NT_x_ MNene. The inset displays the SAED pattern, confirming the hexagonal crystal structure of the MNene. (d) Morphology and (e) height profiles of delaminated V_2_NT_x_ MNene obtained by atomic force microscopy (AFM).

To measure the optical absorbance of synthesized MNene and MXene, UV–vis absorption spectroscopy was performed, and the corresponding spectra are presented in Figure 4a. We observed a distinct maximum absorption peak in the UV region of ∼260 nm for both V_2_NT_x_ MNene and V_2_CT_x_ MXene, which is not present in their MAX phase precursors. While the UV–vis absorption of V_2_CT_x_ MXene has been previously reported in the literature,^32^ we report the optical absorbance V_2_NT_x_ MNene, which exhibits a similar absorption characteristic within the same UV–vis region as V_2_CT_x_ MXene. Furthermore, the absence of a peak around <250 nm indicates that little tono oxidation of the V_2_NT_x_ MNene and V_2_CT_x_ MXene occurred during the etching process.^32^ It is worth noting that this chemical etching process also results in surface functionalization of these materials. To verify this, we turn to FTIR spectroscopy to study the surface termination bond vibrations of V_2_NT_x_ MNene and V_2_CT_x_ MXene, as shown in Figure 4b.

**Figure 4:**
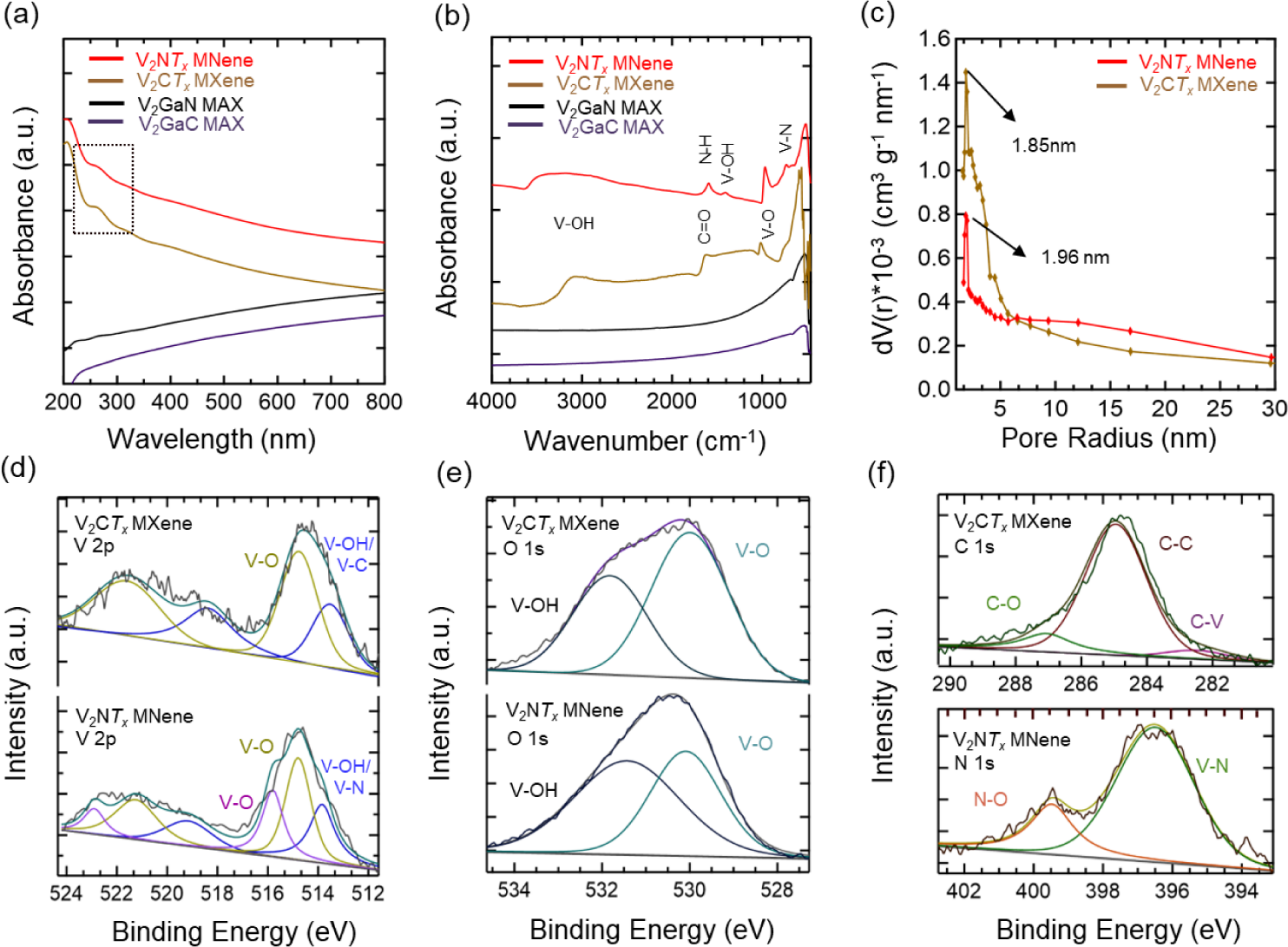
(a) UV–vis absorption spectra of V_2_GaN, V_2_GaC MAX phases, and V_2_NT_x_ MNene and V_2_C*T*_*x*_ MXene. (b) FTIR spectra for V_2_GaN, V_2_GaC MAX phases, and V_2_NT_x_ MNene and V_2_CT_x_ MXene. (c) Pore size distribution of V_2_NT_x_ MNene and V_2_CT_x_ MXene. XPS spectra for (d) V 2p, (e) O 1s, and (f) C 1s and N 1s for the delaminated V_2_NT_x_ MNene and V_2_CT_x_ MXene.

As compared to the MAX phase precursor, there is a significant modification of the synthesized material with the presence of a broad spectrum at 3600–3000 cm^–1^, assigned to the O–H stretching vibration bond, and another peak at ∼1400 cm^–1^, a characteristic of O–H bending, confirming the presence of hydroxyl surface termination group. The peak around 1650 cm^–1^ belongs to the C=O stretching of the V_2_CT_x_ MXene,^42^ while the peak around 1600 cm^–1^ is attributed to the N–H bending of the V_2_NT_x_ MNene.^43^ The presence of a peak at the fingerprint region of ∼ 750 cm^–1^ is an indication of the V–N vibration bond of the V_2_NT_x_ MNene. We also perform N_2_ physisorption measurements to obtain the specific surface area (SSA) and pore size distribution of the synthesized MNene and MXene. After the etching of gallium atoms, the SSA increased from 2.75 m^2^ g^–1^ of the V_2_GaC MAX precursor to 20.68 m^2^ g^–1^ of the V_2_CT_x_ MXene, as determined using the Brunauer–Emmett–Teller (BET) model. In comparison, the SSA of V_2_CT_x_ was higher than the 17.46 m^2^ g^–1^ of V_2_NT_x_ MNene, as shown in the N_2_ adsorption isotherms in Figure S6. This slight decrease in the surface area of V_2_NT_x_ is likely due to the contribution of mesoporous distribution at the ∼6 to 20 nm region of the pore size distribution spectra (Figure 4c), when compared to the V_2_CT_x_ MXene. However, the microporous distribution of the V_2_NT_x_ MNene peaks at ∼1.96 nm, while that of the V_2_CT_x_ MXene peaks at 1.85 nm.

XPS survey spectra were collected for the V_2_GaN MAX phase and the corresponding etched V_2_NT_x_ MNene to assess the elemental composition changes upon etching. The survey data (Figure S7) clearly show a marked reduction in the Ga 3d and Ga 2p peak intensity in the MNene sample compared with the parent MAX phase, confirming the effective removal of gallium during the etching process. High-resolution XPS spectra of the Ga 3d region further confirm this significant decrease in counts per second for the MNene relative to the MAX phase, corresponding to an ∼ 90% reduction in Ga atomic content, indicating substantial gallium etching. To further investigate the chemical state of the synthesized MNene and MXene surfaces, high-resolution XPS analysis was performed. The high-resolution spectrum of V 2p XPS for V_2_NT_x_ MNene shown in Figure 4d was deconvoluted into three distinct peaks with binding energies of ∼513.83, ∼515.07, and ∼515.95 eV. These peaks are attributed to vanadium species bonded to hydroxyl (V–OH) and oxygen (V–O) functional groups, which are indicative of surface oxidation and hydroxylation during the synthesis process. Similarly, the V 2p XPS spectrum for V_2_CT_x_ MXene reveals two fitted peaks at binding energies of ∼513.60 and ∼516.05 eV, corresponding to the presence of V–OH/V–C and V–O on the surface of the V_2_CT_x_ MXene, as reported in previous studies.^39,44,45^ The deconvolution of the O 1s spectra for V_2_NT_x_ MNene confirms the presence of two peaks of V–OH and V–O bonds with binding energies of ∼532.42 and ∼530.04 eV (Figure 4e). This is also seen in the V_2_CT_x_ MXene with binding energies of ∼532.81 and ∼530.03 eV, respectively. The C 1s spectrum of V_2_CT_x_ MXene is shown in Figure 4f in which a peak at ∼282.5 eV is related to the C–V bond inside V_2_CT_x_ MXene. The other two peaks with binding energies of ∼285.1 and ∼287.5 eV are attributed to the C–C and C–O bonds.^46,47^ Furthermore, the deconvoluted N 1s spectrum exhibits two primary peaks at binding energies of ∼397.23 and ∼399.57 eV, which are attributed to V–N and N–O, respectively.^48^

Raman spectroscopy was employed to investigate the molecular structure and transformation of the surface composition of MNene and MXene. Figure S8 shows the Raman spectra of the V_2_GaN and V_2_GaC MAX phases, along with their respective V_2_NT_x_ MNene and V_2_CT_x_ MXene. The MAX phases show distinct Raman peaks at 158, 198, 289, and 407 cm^–1^ respectively.^49,50^ The vibrations at 158 cm^–1^ (E_2g_) and 289 cm^–1^ (E_1g_) correspond to the in-plane and out-of-plane movements of the V atoms. Notably, the sharp Raman peak at 158 cm^–1^ assigned to the E_2g_ mode in the MAX phase disappears upon surface modification and Ga etching, transforming into broader peaks when converted to MNene and MXene. The V_2_NT_x_ MNene and V_2_CT_x_ MXene exhibit characteristic Raman peaks at 240, 364, 482, and 645 cm^–1^. The vibration around 364 cm^–1^ is attributed to the A_1g_ mode, which represents the out-of-plane vibrations of the vanadium atoms, while the peak near 482 cm^–1^ corresponds to the E_2g_ mode, indicative of in-plane vibrations of the vanadium atoms in the V_2_N­(OH)_2_ species. The Raman peak at 645 cm^–1^ suggests the presence of mixed heterogeneous terminal groups (O, F, OH) on both V_2_NT_x_ MNene and V_2_CT_x_ MXene obtained during the synthesis process.^50,51^

The electrochemical performance of the synthesized V_2_NT_x_ MNene electrodes was measured by using a three-electrode configuration in both acidic and alkaline aqueous electrolytes. To begin with, we first observed a large potential window range of −1.2 to 0.6 V vs Hg/Hg_2_SO_4_ for both V_2_NT_x_ and V_2_CT_x_ electrodes in a 1 M acidic H_2_SO_4_ electrolyte (Figure 5a,b) compared to those in the alkaline electrolyte, which had a potential window range of −1.0 to 0.4 V vs Ag/AgCl in 1 M KOH (Figure 5c,d). Water splitting in aqueous electrolytes occurs past ∼1.23 V, which limits the voltage windows and thus the capacity. However, in our study, we notice a large potential window of 1.8 and 1.4 V in acidic and alkaline media. These potential windows can result in increased energy density, making this MNene a promising candidate for high-performance energy storage applications.^52^ The CV curves of the V_2_GaN and V_2_GaC MAX phases (shown in Figure S9) in both acidic and alkaline electrolytes did not show any peaks, indicating the absence of redox activity in the MAX phases. In contrast, the CV curves of the V_2_NT_x_ MNene in acidic media at 2 mV s^–1^ scan rates reveal anodic and cathodic peaks across the potential window, indicating a characteristic charge storage behavior associated with reversible redox reactions. This is in clear contrast to previously reported works for bulk VN material, which exhibits Faradaic redox activities in alkaline electrolytes but not in acidic electrolytes. This redox surface chemistry is attributed primarily to the presence and interaction of hydroxyl ions from the electrolyte with the vanadium surface, forming VN_x_O_y_–OH on the electrode surface and resulting in a pseudocapacitance charge storage mechanism.^5,8,23^ Interestingly, many prior studies have focused primarily on the electrochemical performance of VN materials in alkaline media, as these materials are often considered unstable in acidic environments. To further investigate the nature of the charge storage kinetics, we conducted a b-value analysis based on the scan rate dependence of the peak currents, as shown in Figure S10. The b-values for V_2_NT_x_ MNene in acidic and alkaline electrolytes were determined to be approximately 0.80 and 0.70, respectively. In general, battery-like electrodes are characterized by diffusion-controlled processes, which correspond to a b-value of 0.5, while capacitive and pseudocapacitive electrodes are associated with surface-controlled processes, with a b-value of 1.^53^ The b-values between 0.5 and 1 are assumed to represent a transition area from battery-like to capacitive, with values between 0.85 and 1 indicative of dominant surface reactions.^53^ Considering these analyses, we suspect that the V_2_NT_x_ MNene electrode kinetics are influenced by both surface reactions and diffusion-controlled reactions, with the latter presumably playing a dominant role.^53–55^

**Figure 5:**
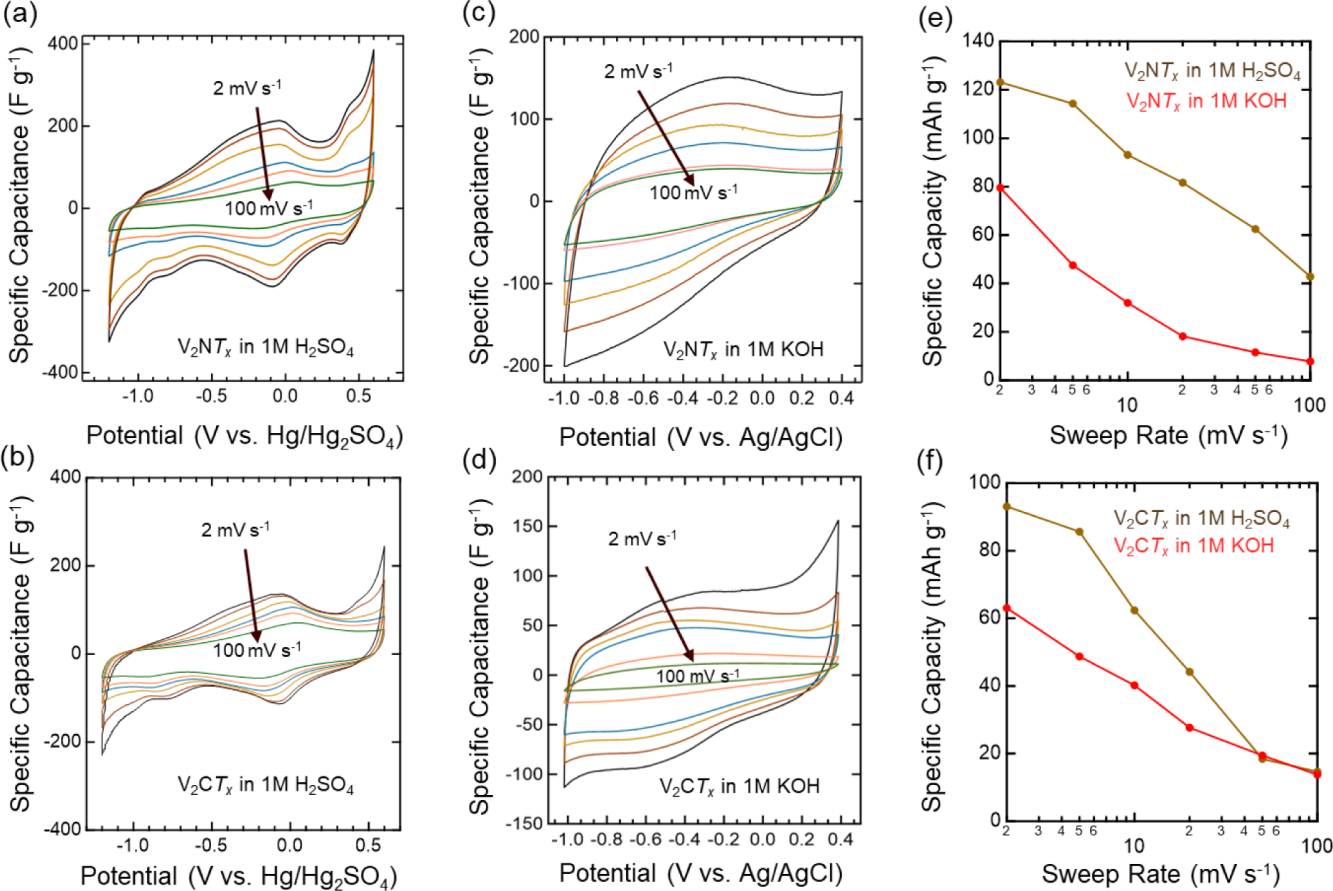
Electrochemical measurements of V_2_NT_x_ MNene and V_2_CT_x_ MXene, etched using the O_2_-MSE method. CV curves of the V_2_NT_x_ MNene and V_2_CT_x_ MXene electrodes in (a,b) 1 M H_2_SO_4_ and (c,d) 1 M KOH electrolytes. CV was performed at different scan rates (from outside to inside: 2, 5, 10, 20, 50, and 100 mV s^–1^). Comparison of the specific capacity of (e) V_2_NT_x_ MNene and (f) V_2_CT_x_ MXene as a function of scan rate in 1 M H_2_SO_4_ and 1 M KOH electrolytes, obtained from the anodic CV scans.

However, our results challenge this assumption, demonstrating that V_2_NT_x_ MNene exhibits fast, reversible redox reactions across the potential window in acidic electrolytes. The V_2_NT_x_ electrode in alkaline media does not follow the conventional charge storage mechanisms previously reported for VN materials under similar conditions. We attribute the unique electrochemical behavior observed in acidic media to the surface chemistry of V_2_NT_x_ MNene, which is enriched with polar functional groups. Our FTIR and XPS data (Figure 4b,d-f) confirm that the basal plane of V_2_NT_x_ MNene is decorated with surface terminations such as the O and OH groups. These hydroxyl functional groups significantly modify the electrochemical activity of the material, influencing charge storage behavior in both acidic and alkaline conditions.

The specific capacity of V_2_NT_x_ MNene was determined from the CV curves at varying scan rates of 2, 5, 10, 20, 50, and 100 mV s^–1^, as shown in Figure 5e. At a low scan rate of 2 mV s^–1^, the maximum specific capacity recorded for V_2_NT_x_ MNene in the 1 M H_2_SO_4_ electrolyte was 123 mAh g^–1^, which is higher than 80 mAh g^–1^ obtained in the 1 M KOH electrolyte. This is also in contrast to bulk VN, which exhibits higher capacity in alkaline media.^56–58^ The superior capacity of V_2_NT_x_ MNene in acidic electrolytes suggests that the presence of oxygen and hydroxyl surface terminations alters its electrochemical behavior, enabling enhanced redox activity and charge storage in H_2_SO_4_, unlike bulk VN, which relies on hydroxyl ion interactions under alkaline conditions. A similar behavior was observed with the synthesized V_2_CT_x_ MXene electrode, as illustrated in Figure 5f, which further indicates the influence of the surface termination group in enhancing a specific type of mechanism in different electrolyte environments, regardless of the type of MXene used. For both acidic and alkaline electrolytes, an inverse relationship between the scan rate and specific capacity was observed. At lower scan rates, electrolyte ions have sufficient time to intercalate into and deintercalate from the electrode material, enabling maximum interaction with active sites. However, at higher scan rates, the limited time available for ion migration restricts the intercalation and deintercalation process on the MNene and MXene structure, thereby resulting in limited charge storage.^59^ However, in 1 M H_2_SO_4_, the specific capacity of V_2_CT_x_ MXene at a scan rate of 2 mV s^–1^ was 93 mAh g^–1^, which is lower than that of V_2_NT_x_ MNene. The Coulombic efficiency (CE) of V_2_NT_x_ MNene in acidic and alkaline environments, shown in Figure 6a, is approximately 96% and 92%, respectively, after 10,000 cycles, whereas that for the V_2_C*T*_*x*_ MXene is around 94% and 91% (Figure 6b). The CE for both materials stays relatively constant after thousands of cycles, indicating good stability. However, we note that the value does not start at 100%. The underlying reason for this is presently unclear to us, but we suspect it could be due to factors such as parasitic reactions between the electrolyte and the MNene interface, electrolyte decomposition, high series resistance, or insufficient electronic conductivity of the electrodes.^53,54,60,61^

**Figure 6:**
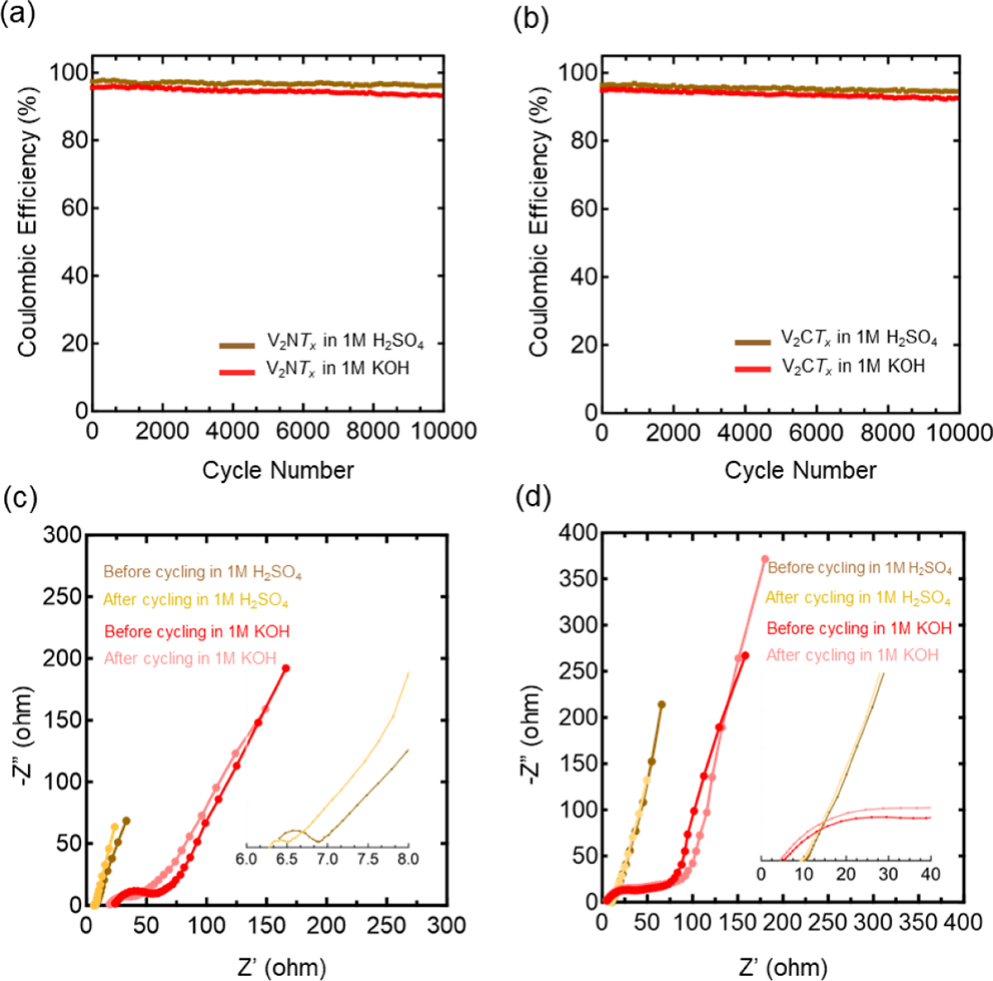
Coulombic efficiency as a function of CV cycle for (a) V_2_NT_x_ MNene and (b) V_2_CT_x_ MXene. Nyquist plots of (c) V_2_NT_x_ MNene and (d) V_2_CT_x_ MXene in 1 M acidic H_2_SO_4_ and 1 M KOH electrolytes, comparing impedance before and after prolonged cycling. The inset shows a magnified view of the high-frequency region.

To further investigate the electrochemical behavior of V_2_NT_x_ MNene and V_2_CT_x_ MXene electrodes, electrochemical impedance spectroscopy (EIS) was performed before and after 10000 CV cycles over a frequency range of 200 kHz to 10 mHz with a 10 mV amplitude in both acidic and alkaline electrolytes. The Nyquist plots (Figure 6c,d) show that the systems deviate from purely capacitive charge storage, consistent with the b-value analysis. Unlike the sharp 45° transition followed by a steep low-frequency rise reported by Diard et al., which indicates full accessibility of active sites, our Nyquist plots lack this feature, suggesting that some active sites are not accessed within the measurement time scale.^62^ This may explain why the CE of both materials is below 100%. Two high-frequency resistances are evident: the *x*-axis intercept, attributed to the equivalent series resistance (ESR), and the semicircle diameter, attributed to either interfacial impedance or charge-transfer resistance (*R*_ct_). The decrease of this resistance after cycling indicates it is most likely *R*_ct_, since interfacial impedance is typically voltage- and cycle-independent, and when it changes, it tends to increase rather than decrease. ESR and *R*_ct_ values from the Randles circuit fitting are summarized in Table S2. For V_2_NT_*x*_ MNene, *R*_ct_ decreased from 0.51 to 0.23 Ω in an acidic electrolyte and from 25.94 to 15.80 Ω in an alkaline electrolyte. For V_2_CT_x_ MXene, *R*_ct_ dropped from 0.42 to 0.34 Ω in an acidic electrolyte and from 34.07 to 30.92 Ω in an alkaline electrolyte. The high-frequency *R*_ct_ likely arises from electrostatic double-layer charge storage, with similar values for both materials in an acidic electrolyte and substantially lower values than in an alkaline electrolyte.^63^ Given their comparable surface areas, these trends likely reflect the size difference between H^+^ and OH^–^ ions. ESR values are also much lower in an acidic electrolyte, consistent with the high mobility of H^+^ ions via Grotthuss conduction, where protons “hop” between water molecules, compared to the slower transport of OH^–^ ions.^64,65^ While these analyses shed light on interfacial processes, the fundamental mechanisms of charge storage responsible for the observed CV behaviors must be further investigated. Future operando FTIR spectroelectrochemical studies at the electrode–electrolyte interface will be essential to elucidate these underlying mechanisms.

## Conclusion

In conclusion, we synthesized layered vanadium MNene using a modified O_2_-MSE method that we developed for titanium-based MNenes, showcasing its electrochemical performance, stability, and surface chemistry. The O_2_-MSE method not only enabled the synthesis of V_2_NT_x_ MNene but also demonstrated its versatility by synthesizing the corresponding carbide phase, V_2_CT_x_. Different chemical and physical analyses confirm the phase purity, layered morphology, crystallinity, surface area, and chemical composition of the synthesized V_2_NT_x_ and V_2_CT_x_ materials. The synthesized MNene exhibited enhanced electrochemical performance, with a capacity of 123 mAh g^–1^ in acidic electrolyte and outstanding long-term cycling stability compared to its carbide counterpart. This enhanced performance is attributed to the unique O and/or OH surface groups, which facilitate reversible redox reactions in acidic environments. These findings establish V_2_NT_x_ MNene as a promising candidate for energy storage devices, particularly for applications requiring long-term stability.

## Experimental Section

### Materials

Vanadium (Alfa Aesar, 325 mesh, 99.5%), carbon (Alfa Aesar, APS 2–15 μm, 99.999%) and gallium flakes (Alfa Aesar, >99%) were used. Sodium fluoride (NaF), lithium fluoride (LiF), and potassium fluoride (KF) were purchased from Sigma-Aldrich. Activated carbon, 5% poly­(vinylidene fluoride) (PVDF) in *N*-methyl-2-pyrrolidone (NMP), and carbon black were also utilized. Sulfuric acid (H_2_SO_4_, ACS reagent, >98% purity) and potassium hydroxide (KOH, ACS reagent, >99% purity) were used as electrolytes for electrochemical performance measurements. All the chemicals were used as received without further purification. Deionized water (DI) was used for acid washing and rinsing of the V_2_NT_x_ MNene and V_2_CT_x_ MXene.

## Synthesis of V_2_GaN and V_2_GaC MAX Precursors

The detailed synthesis process for V_2_GaN and V_2_GaC MAX phases has been previously reported by the Birkel group.^17^ In brief, the powdered precursors—vanadium (Alfa Aesar, 325 mesh, 99.5%), carbon (Alfa Aesar, APS 2–15 μm, 99.999%), or vanadium nitride (VN, synthesized according to Vaidhyanathan^66^)—were thoroughly mixed using an agate mortar inside an argon-filled glovebox. Afterward, the powder mixture was loosely combined with gallium flakes (Alfa Aesar, >99%) and pressed into a dense pellet (*d* = 10 mm, 3 t, 5 s), which was then transferred into a fused silica ampule. The molar precursor ratios were 2 V: 1 Ga: 1 C for the V_2_GaC phase, while the V_2_GaN synthesis followed a ratio of 1 V: 1 Ga: 1 VN. Heat treatments were conducted in a chamber furnace (Snoltherm) at 1000 °C with a holding time of 15 h for the carbide and 30 h for the nitride.

## Synthesis of V_2_NT_x_ MNene and V_2_CT_x_ MXene

The vanadium nitride (V_2_NT_x_) MNene and vanadium carbide (V_2_CT_x_) MXene were synthesized by selectively etching the Ga atom from their MAX precursors (V_2_GaN and V_2_GaC) using the oxygen-assisted molten salt etching method. First, LiF (Alfa Aesar, 325 mesh, 98.5%), NaF (Alfa Aesar, 99%), and KF (Alfa Aesar, 99%) were mixed in a molar ratio of 29:12:59%, after which the mixture was then combined with the V_2_GaN MAX in a 1:2 ratio and ground for about 10 min to achieve a uniform blend. The resulting mixture was transferred into a crucible and placed inside a quartz tube furnace (ATS Series 3210). The furnace was heated to 550 °C at a controlled ramp rate of 5 °C/min and maintained at this temperature for 30 min under continuous argon flow (Ultra High Purity, Airgas) at a flow rate of 400 mL/min. After this period, the argon flow was discontinued, and the 3/16″ tubing of the furnace outlet was exposed to air for approximately 30 to 45 min (for V_2_CT_x_ MXene, the exposure was limited to 30 min). The furnace was then sealed for an additional 1 h before being gradually cooled to room temperature. The molten salt-treated MAX (MST-MAX) was acid-washed with deionized water (18.2 MΩ·cm, Milli-Q) to remove residual complex salt mixtures and obtain the corresponding multilayered MNene and MXene. The washing process continued until a neutral pH was achieved, after which the material was vacuum-filtered. This material is considered multilayered MNene and MXene. To achieve few-layer and single-layer MNene and MXene, the acid-washed material was dispersed in deionized water and subjected to sonication for about 30 min to separate the MNene and MXene sheets. After sonication, the mixture was left undisturbed for about 45 min. The supernatant, which contained the delaminated MNene and MXene nanosheets, was then carefully filtered, dried at 50 °C overnight in a vacuum oven, transferred into a vial, and stored in an inert environment.

## Physical Characterization

The crystal structure of the synthesized materials was analyzed by powder X-ray diffraction (XRD) using a Rigaku MiniFlex 6G diffractometer. The measurements were conducted at 40 mA and 45 kV using Cu Kα radiation (λ = 0.15418 nm) with a scan rate of 2° min^–1^ over a 2θ range of 3° to 70° with a step size of 0.03°. Surface morphology and elemental composition were investigated by using scanning electron microscopy (SEM, JSM-IT200) coupled with energy-dispersive X-ray (EDX) spectroscopy to confirm the structural and compositional integrity of the synthesized materials. The layered structure and morphology of the materials were further examined using a transmission electron microscope (TEM; Thermo Fisher Titan Themis3 300) to obtain high-resolution imaging and analysis of the MNene nanosheets, operated at an acceleration voltage of 300 kV. The samples for TEM were prepared by drop-casting diluted supernatants of the V_2_NT_x_ MNene onto an amorphous carbon film supported by a copper grid. The interlayer spacing of V_2_NT_x_ MNene was estimated by using ImageJ software from TEM images. Fourier-transform infrared spectroscopy measurements were performed using a Bruker INVENIO-R with a diamond ATR to measure the absorbance over a wavelength range of 400 to 4000 cm^–1^. The specific surface area and pore structure were determined via N_2_ physisorption measurements by using a Quantachrome Autosorb-iQ analyzer. The Brunauer–Emmett–Teller (BET) method was applied to calculate the surface area after degassing the samples under vacuum at 80 °C for 6 h. Raman spectroscopy was carried out using a Renishaw inVia Qontor with a 532 nm laser, 1800 lines/mm grating, and a 50× long objective lens. The optical absorption properties of the synthesized V_2_NT_x_ MNene and V_2_CT_x_ MXene were evaluated using a Shimadzu UV-1800 UV–vis spectrophotometer, scanning absorbance over the 200–800 nm wavelength range. Atomic force microscopy (AFM) was conducted in tapping mode using a Dimension Icon AFM (Bruker) instrument to assess the thickness of the V_2_NT_x_ MNene and V_2_CT_x_ MXene. For sample preparation, a dilute dispersion of V_2_NT_x_ MNene and V_2_CT_x_ MXene (∼0.01 mg mL^–1^) in isopropanol was prepared, drop-cast onto a mica substrate, and then dried under vacuum before analysis. X-ray photoelectron spectroscopy (XPS) was used to analyze the surface chemistry and elemental states of V_2_NT_x_ MNene and V_2_CT_x_ MXene. The measurements were conducted using an Omicron XPS system with an Argus detector at the Texas A&M University Materials Characterization Facility (RRID: SCR_022202). XPS data were acquired using a monochromatic Al Kα source (hv = 1486.61 eV) at 13 kV and 100 W, with a constant analyzer energy of 40 eV and a dwell time of 0.05 s. Survey scans were performed using a step size of 1 eV and a dwell time of 50 ms. High-resolution scans of the V 2p, N 1s, O 1s, Ga 3d, and C 1s regions were performed using step sizes of 100 meV and dwell times of 300 ms. Three spectra were averaged for accuracy. X-ray control settings included a 15 mA emission current and 15 kV anode current (225 W power), while the CN10 neutralizer operated at 10 μA with a 2 eV beam energy for charge compensation.

## Electrochemical Measurements

The electrochemical performance of the synthesized materials was evaluated in a three-electrode plastic Swagelok T-cell by using a Biologic VSP 300 potentiostat. All measurements were recorded at room temperature. Glassy carbon rods were used as current collectors, while the counter electrode comprised 95 wt % activated carbon (YP-50) and 5 wt % polytetrafluoroethylene (PTFE). Ag/AgCl in saturated KCl and Hg/Hg_2_SO_4_ in saturated K_2_SO_4_ were used as the reference electrodes. The working electrode was prepared using synthesized MNene and MXene, carbon black, and poly­(vinylidene fluoride) (PVDF) in a weight ratio of 85:10:5, which were combined and blended using *N*-methyl-2-pyrrolidone (NMP) solvent. A Celgard 3401 separator was used between the working and counter electrodes in the three-electrode system. The electrodes were tested in aqueous 1 M KOH and 1 M H_2_SO_4_ electrolytes. Both electrolytes were bubbled with argon for 1 h for degassing before usage. The open-circuit voltage (OCV) was applied and held for 10 min before starting any measurements. Cyclic voltammetry (CV) was performed at scan rates ranging from 2 to 100 mV s^–1^. The Nyquist plot obtained from electrochemical impedance spectroscopy (EIS) was measured in a frequency range from 200 kHz to 10 mHz at an amplitude of 10 mV, both before and after cycling. The specific capacitance (F g^–1^) values were calculated using

$$C=\frac{\int I\left(V\right)dV}{m\cdot \Delta V\cdot v}$$

where *C* is the specific capacitance, *I*(*V*) *dV* is the area under the CV curve, *m* is the mass of the working electrode, Δ*V* is the potential window, and *v* is the scan rate.

The specific capacity (*Cs*) was calculated using

$$Cs=\frac{\int I\left(V\right)dV}{3.6m\cdot v}$$

## Supplementary Material



## Notes

XRD patterns of V_2_GaN and V_2_GaC MAX phases, oxygen-assisted molten salt-treated V_2_NT_x_ MNene and V_2_CT_x_ MXene, and delaminated V_2_NT_x_ MNene and V_2_CT_x_ MXene; SEM images of multilayer and delaminated V_2_NT_x_ MNene and V_2_CT_x_ MXene, along with EDS elemental mapping; TEM images of multilayer and delaminated V_2_NT_x_ MNene, including SAED patterns confirming the hexagonal crystal structure; UV–vis absorption spectra of V_2_GaN, V_2_GaC MAX phases, and corresponding V_2_NT_x_ MNene and V_2_CT_x_ MXene; FTIR spectra confirming functional groups on V_2_NT_x_ MNene and V_2_CT_x_ MXene; XPS spectra detailing V 2p, O 1s, C 1s, and N 1s for both V_2_NT_x_ MNene and V_2_CT_x_ MXene; N_2_ physisorption analysis illustrating pore size distribution and surface area; electrochemical data including cyclic voltammetry (CV) and capacitance curves; Nyquist plots from electrochemical impedance spectroscopy (EIS) before and after cycling; and long-term cycling stability results for V_2_NT_x_ MNene and V_2_CT_x_ MXene in 1 M H_2_SO_4_ and 1 M KOH electrolytes (PDF)
