# Supplementary material for: Synthesis, Characterization, and Electrochemical Behavior of Layered Vanadium Nitride MXene
Source: ACS Nano. 2025 Sep 20;19(38):34438–48. doi: 10.1021/acsnano.5c14516 (PMC12490019; doi:10.1021/acsnano.5c14516)
Supplement: Supplementary file 1 [file nn5c14516_si_001.pdf]

Supporting Information

Synthesis, Characterization, and Electrochemical Behavior of Layered Vanadium Nitride MXene

Bright Ngozichukwu[1], Niels Kubitza[2], Laura Hoagland[1], Christina S. Birkel[2,3]*, Abdoulaye Djire[1,4]*

[1]Artie McFerrin Department of Chemical Engineering, Texas A&M University, College Station, TX 77843, USA

[2]Department of Chemistry and Biochemistry, Technische Universitat Darmstadt, Darmstadt, 64287, Germany

[3]School of Molecular Sciences, Arizona State University, Tempe AZ-85282, USA

[4]Department of Materials Science & Engineering, Texas A&M University, College Station, TX 77843, USA

*Corresponding Authors: Christina.Birkel@asu.edu adjire@tamu.edu

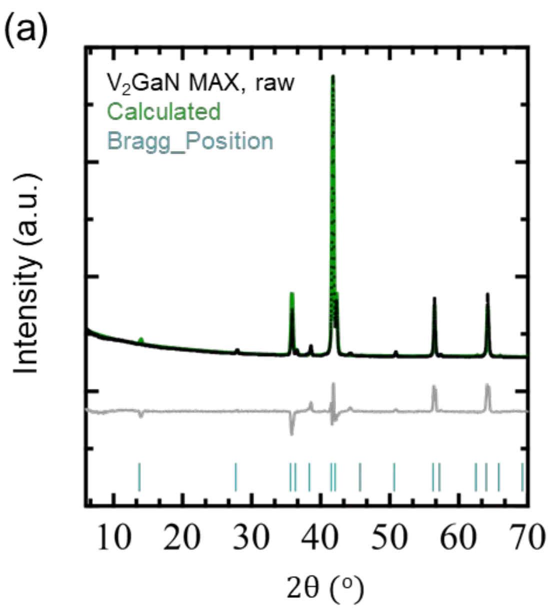

Figure S1. Rietveld refinement analysis for (a) V_2GaN and (b) V_2GaC MAX phases. Experimental XRD patterns are shown in black, calculated refinement data in green and bragg position reference patterns in teal.

Table S1. Refined lattice parameters of the V_2GaN and V_2GaC MAX phases obtained from Rietveld analysis.

MAX Phase	V_2GaN	V_2GaC
Spacegroup	$P6_3/mmc$	$P6_3/mmc$
Lattice parameter /Å	a = 2.9079(2)<br>c = 12.8759(8)	a = 2.9361(3)<br>c = 12.8651(2)
Cell volume/ Å^3	94.29(2)	96.05(2)
Background order	10	10
R_p	6.06	8.28
R_{wp}	11.04	13.47
R_{exp}	1.23	1.15
GOF	8.99	11.67

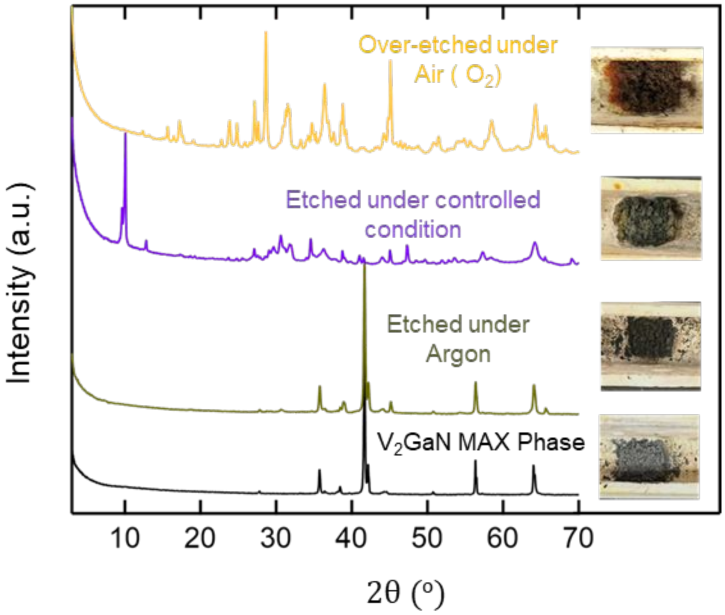

Figure S2. X-ray diffraction (XRD) pattern of the molten salt-etched V_2GaN MAX phase under different etching conditions. When etched under continuous Argon flow (brown diffraction pattern), the prominent (104) peak at approximately $2\theta = 42°$ remains, indicating minimal or no significant etching of the MAX phase. Exposure to air during the entire etching process (yellow diffraction pattern) results in oxidation of the material due to over-etching. However, under a controlled oxygen environment (purple diffraction pattern), minimal oxidation is observed, with the disappearance of the (104) peak at $2\theta = 42°$, indicating the transformation of the MAX phase precursor. Additionally, a noticeable color change is evident, as shown in the photographic images, from the light grey appearance of the initial V_2GaN MAX to a dark green product after the oxygen-assisted molten salt etching (O_2-MSE) process.

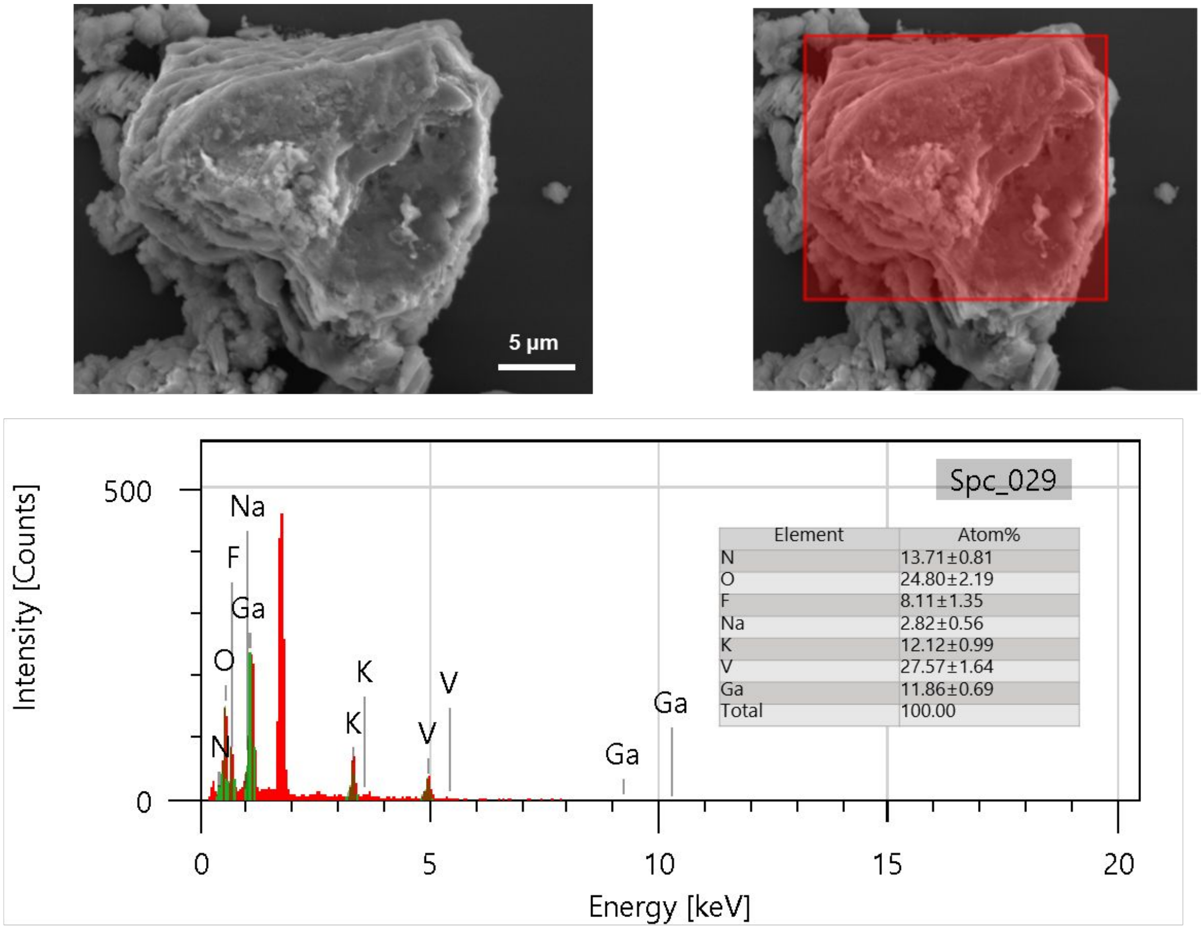

Figure S3. Scanning Electron Microscopy (SEM) image and Energy Dispersive X-ray Spectroscopy (EDS) analysis of the molten salt-etched V_2GaN-salt sample immediately removed from the furnace and before further downstream treatment. The SEM micrograph reveals distinct morphological changes induced by the etching process. The presence of elements such as N, K, F, and Ga is expected due to the synthetic procedure and will be removed upon acid washing.

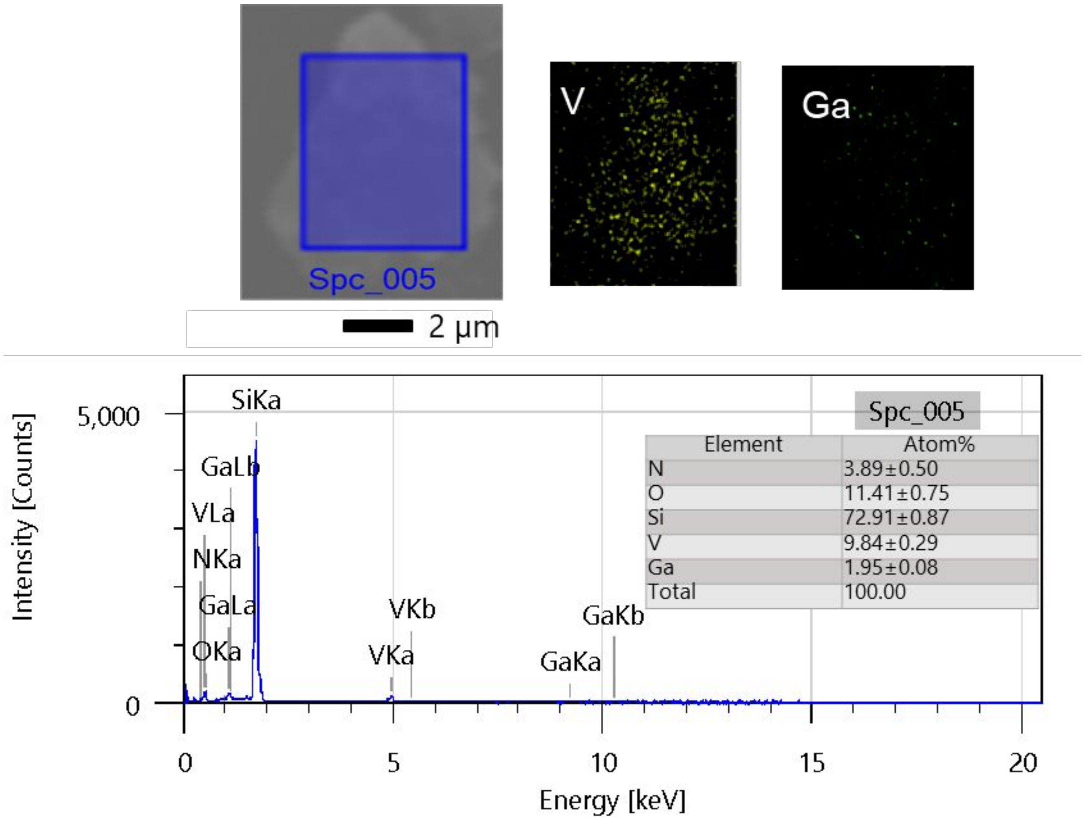

Figure S4. Scanning Electron Microscopy (SEM) image with Energy Dispersive X-ray Spectroscopy (EDS) mapping and analysis of the post-washed V_2NT_x MNene. The results show effective removal of residual salts and a significant reduction in gallium content, as evidenced by both EDS mapping and compositional analysis, confirming Ga etching via the molten salt method.

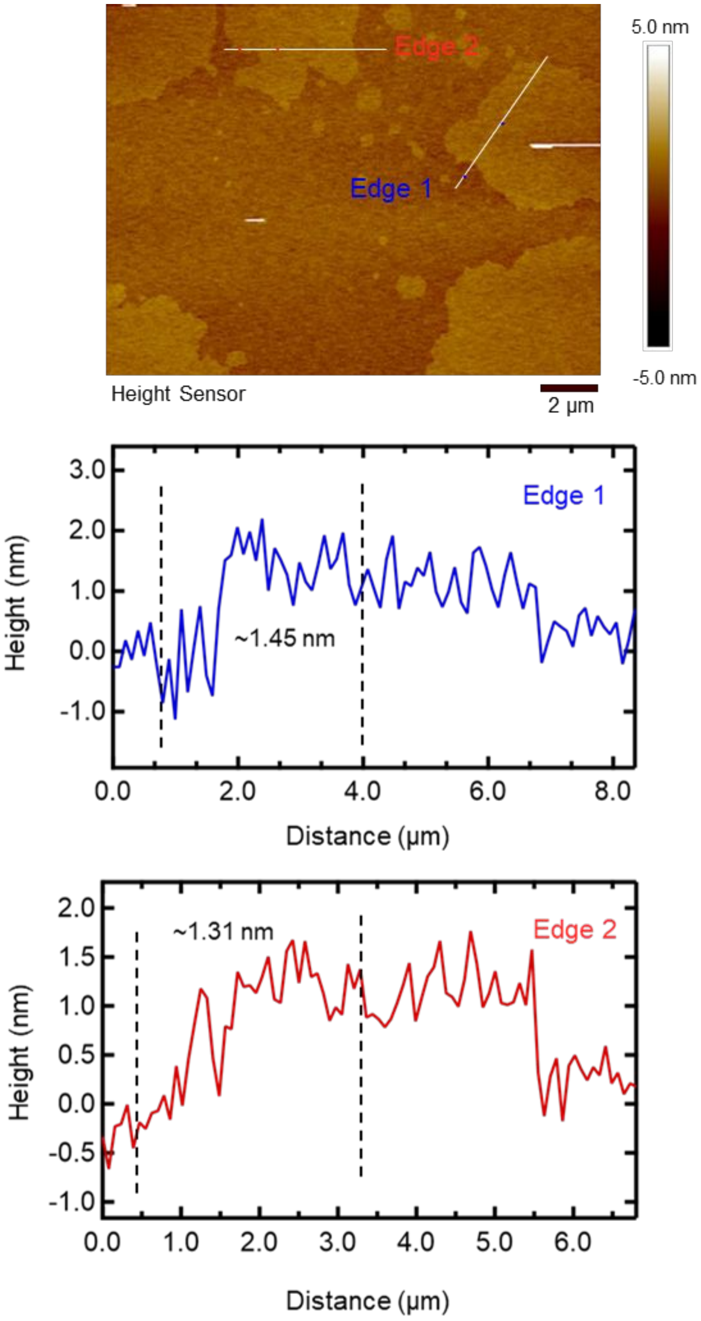

Figure S5. Atomic force microscopy (AFM) of V_2CT_x MXene, showing the surface topography and thickness measurement, with an average thickness of ~1.38 nm, indicating the formation of few-to-single layer MXene sheets.

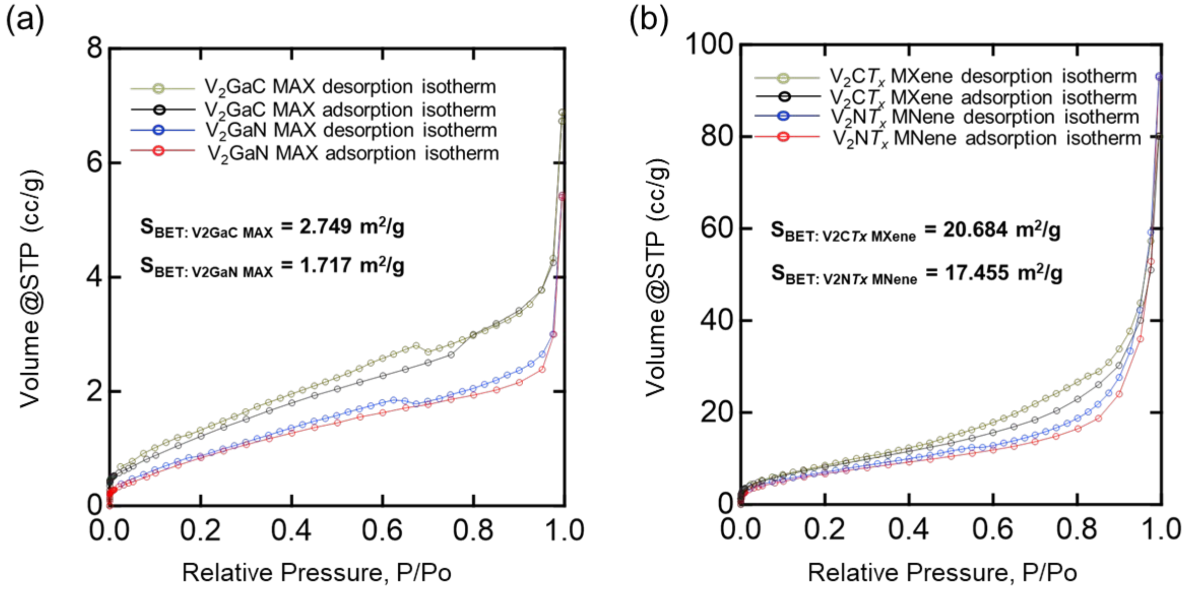

Figure S6. Adsorption and desorption isotherms of (a) V_2GaN and V_2GaC MAX (b) V_2NT_x MNene and V_2CT_x MXene.

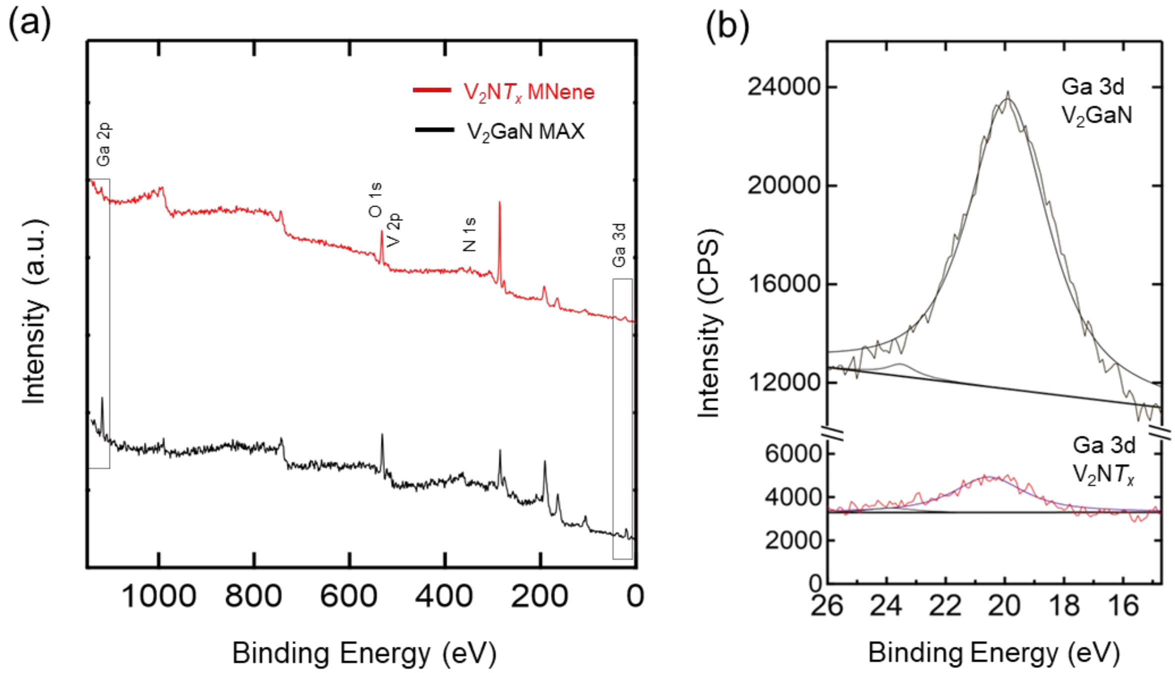

Figure S7. (a) X-ray photoelectron spectroscopy (XPS) survey spectra of V_2GaN MAX phase and the corresponding V_2NT_x MNene, showing the composition changes after etching. The significant reduction in Ga 3d and Ga 2p peak intensity in the MNene sample confirms the etching of gallium from the parent MAX phase. (b) High-resolution XPS spectra of V_2GaN and V_2NT_x in the Ga 3d region clearly show the decreased Ga peak intensity in the MNene, indicating gallium etching during the synthesis process.

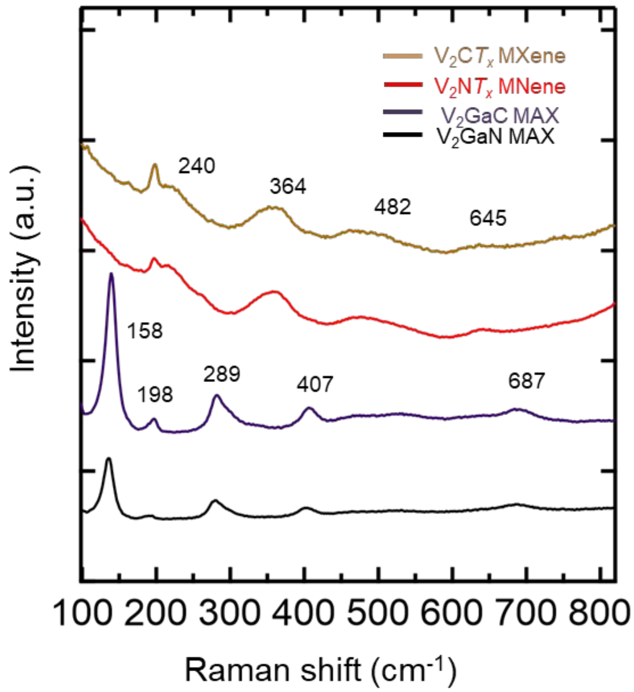

Figure S8: Raman spectra of V_2GaN and V_2GaC MAX phases, along with their corresponding V_2NT_x MNene and V_2CT_x MXene.

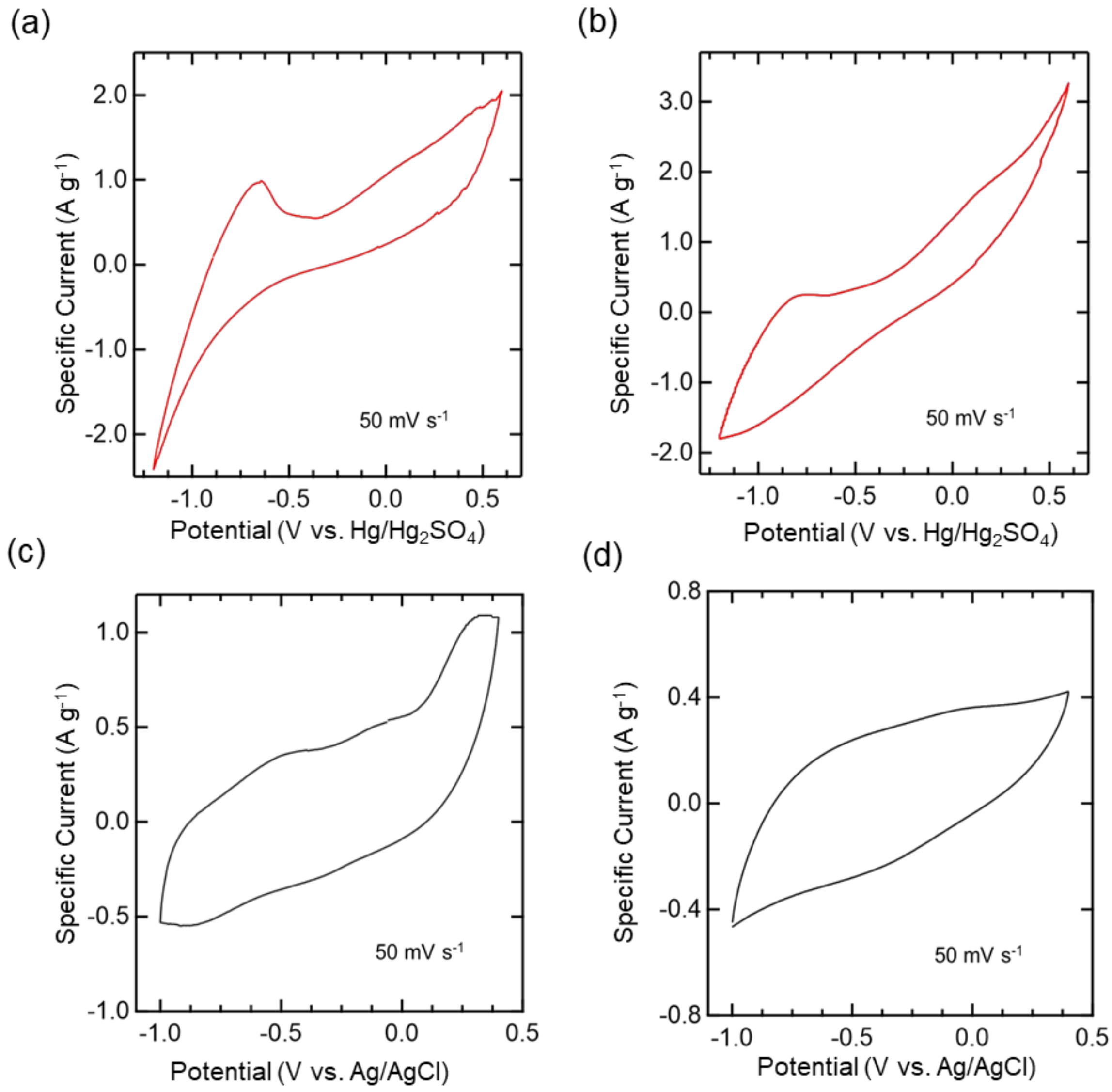

Figure S9. Cyclic voltammetry of (a) V_2GaN MAX Phase and (b) V_2GaC MAX Phase in 1M H_2SO_4 acidic electrolyte and (c) V_2GaN and (d) V_2GaC MAX Phase in 1M KOH electrolyte.

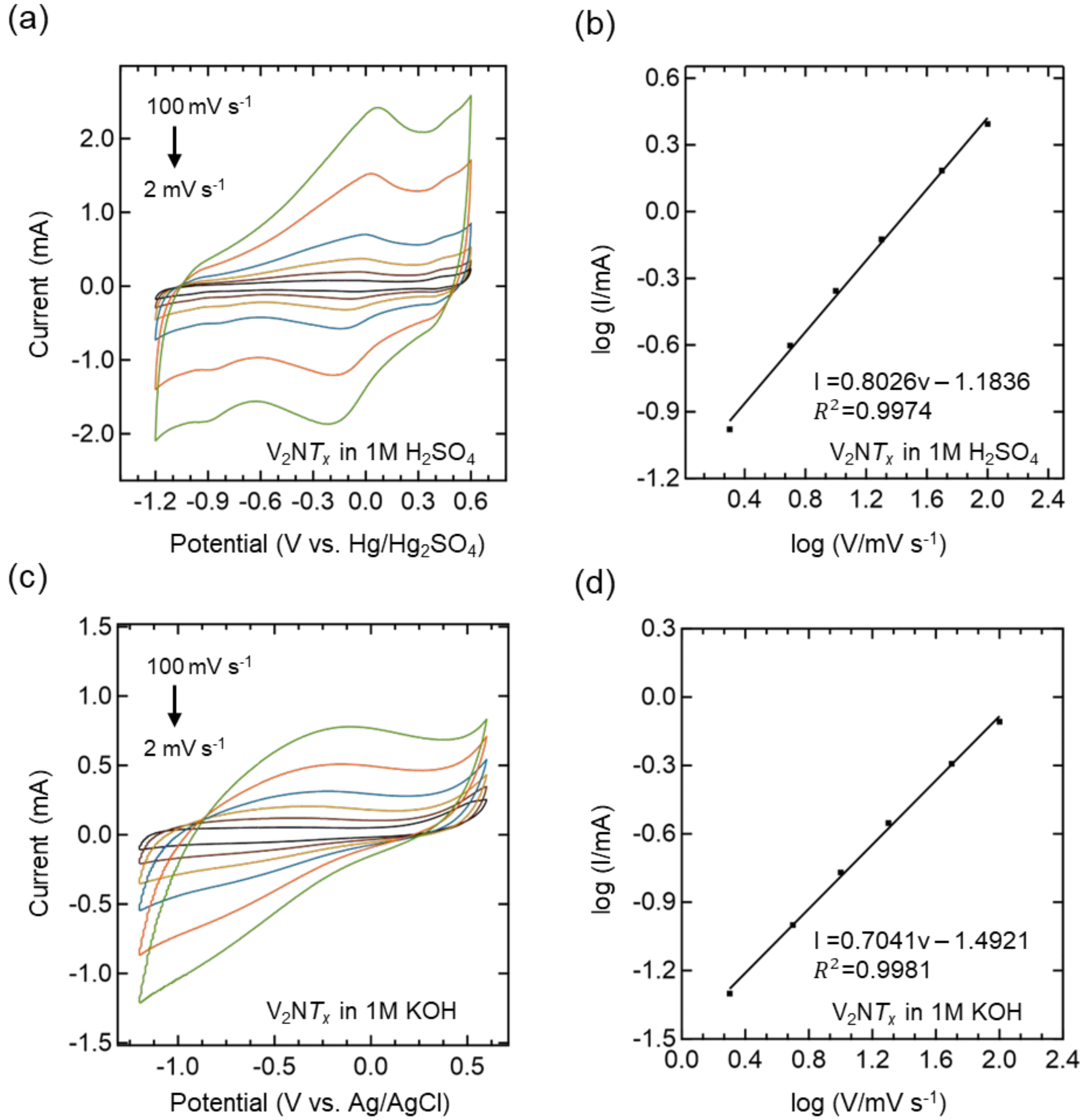

Figure S10. Cyclic voltammetry for (a) V_2NT_x MNene, and (C) V_2CT_x MXene electrode. Linear fitting of log(*i*) versus log(*v*) for anodic peaks of (b) V_2NT_x MNene, and (d) V_2CT_x MXene electrode.

Table S2. Charge transfer resistance (R_{ct}) and Equivalent series resistance (ESR) for V_2NT_x MNene and V_2CT_x MXene, before and after cycling. Fitting was performed using a Randles circuit in the high-frequency region of all samples.

Electrode	Electrolyte	R_{ct} (Ω) Before	R_{ct} (Ω) After	ESR (Ω) Before	ESR (Ω) After
V_2NT_x	H_2SO_4	0.51	0.23	6.31	6.28
	KOH	25.94	15.80	30.03	25.32
V_2CT_x	H_2SO_4	0.42	0.34	10.01	10.00
	KOH	34.07	30.92	6.45	5.47
